# *Trichoderma* spp. from Misiones, Argentina: effective fungi to promote plant growth of the regional crop *Ilex paraguariensis* St. Hil

**DOI:** 10.1080/21501203.2019.1606860

**Published:** 2019-04-22

**Authors:** Ana Clara López, Adriana Elizabet Alvarenga, Pedro Darío Zapata, María Flavia Luna, Laura Lidia Villalba

**Affiliations:** a Laboratorio de Biotecnología Molecular, Instituto de Biotecnología Misiones “Dra. Maria Ebe Reca”, Facultad de Ciencias Exactas, Químicas y Naturales, Universidad Nacional de Misiones, CONICET, Posadas, Argentina; b Centro de Investigación y Desarrollo en Fermentaciones Industriales (CINDEFI), CCT-La Plata CONICET, Departamento de Química, Facultad de Ciencias Exactas, Universidad Nacional de La Plata, La Plata, Argentina

**Keywords:** Trichoderma, Ilex paraguariensis, plant growth promotion, biological control

## Abstract

*Ilex paraguariensis* St. Hil (yerba mate) is an important crop in the north of Argentina, mainly in Misiones province. The application of *Trichoderma* as a biocontroller and biofertilizer can replace or reduce the use of agrochemicals, decreasing the negative ecological impact. In this research, we evaluated *in vitro* and *in vivo* antagonistic and plant growth promoting (PGP) properties of *Trichoderma* species isolated from different regions of Misiones province. Dual culture assays of *Trichoderma* against phytopathogenic fungi associated with yerba mate showed that *T. stilbohypoxyli* LBM 120 was the most effective antagonist, inhibiting in more than 75% all phytopathogen growth. *Trichoderma atroviride* LBM 112 and *T. stilbohypoxyli* LBM 120 were positive on endoglucanase, protease, chitinase, siderophore production, and phosphate solubilisation showed the best biological control agents and PGP properties. The PGP properties of *Trichoderma* spp. evaluated *in vivo* on yerba mate seedlings showed that *T. atroviride* LBM 112, *T. stilbohypoxyli* LBM 120, and *T. koningiopsis* LBM 219 enhanced plant dry weight over 47% in total and 24% in the aerial part. Moreover, *T. koningiopsis* LBM 219 increased root dry weight 25% in contrast with *in vitro* controls. In conclusion, native *Trichoderma* strains could be a sustainable solution to improve yerba mate yield.

## Introduction

*Ilex paraguariensis* St. Hill., a species of the family Aquifoliaceae, is a native tree of the Atlantic Forest in northeastern Argentina, Paraguay, and South Brazil. This tree is a very valuable regional crop because its leaves are processed into a traditional beverage called mate (Bergottini et al. 2015). The features and ecological conditions suitable for its successful growth are met in the aforementioned countries. Moreover, Argentina is the world’s largest producer of this tree; the production takes place mainly in Misiones and Corrientes provinces. Unfortunately, about 50% of the cultivated area is under degradation process. This can be associated essentially to the longevity of the plants, but there are also other factors such as destructive soil management, material with low genetic quality, inadequate harvest, and incorrect pruning, among others (Prat Kricun and Belingheri 2003). In the management of yerba mate crops, it is currently recommended to adopt conservationist practices such as zero tillage (using herbicides) and the introduction of plant species companions as green roofs or native tree species (Eibl et al. 2000; Prat Kricun and Belingheri 2003; Ilany et al. 2010).

The growing increase of the planted area has unleashed epidemic pests and diseases due to the accumulation of susceptible hosts, both on the field and under controlled conditions on a greenhouse. Damping off is one of the major phytosanitary problems undergone by this crop, causing productivity losses of around 30% (Poletto et al. 2006). This disease is commonly caused by several species of *Fusarium* sp., Pythium sp., and *Rhizoctonia* sp. To reduce the use of chemical products, it is recommended to give priority to cultural and biological methods of pest control. The use of fungal strains of the genus *Trichoderma* to control plant diseases is one of the most promising alternatives to the use of chemical fungicides. Furthermore, it has been reported that it has a stimulating effect on the growth of crops such as tomato, bean, cucumber, and others (Gravel et al. 2007; Hoyos-Carvajal et al. 2009).

The aim of this work was to evaluate the biological control capacity *in vitro* and the plant growth promoting (PGP) properties of four *Trichoderma* strains previously isolated from soil of different yerba mate cultivated regions in Misiones province. Antagonist dual assays, production of diffusible and volatile metabolites of *Trichoderma* strains, were performed. The enzymes presumably involved on the antagonist activity of these fungi, such as lipase, endoglucanase, and protease, were also evaluated. The PGP activity was assessed both *in vitro* through the phosphate solubilisation capacity and siderophore production and *in vivo* by inoculation of a spore suspension of *Trichoderma* on yerba mate plants under greenhouse conditions.

## Materials and methods

### Organism and maintenance

The strains used in this study belong to the collection of the Institute of Biotechnology Misiones and were named LBM. The *Trichoderma* strains used were *T. atroviride* LBM 112, *T. koningiopsis* LBM 116, *T. stilbohypoxyli* LBM 120, and *T. koningiopsis* LBM 219.

The phytopathogenic fungi assayed were *Colletotrichum gigasporum* LBM 183, *Fusarium oxysporum* LBM184, *Alternaria destruens* LBM 186, *Phoma destructive* LBM189, *Phoma* sp. LBM 207, and *Pilidium concavum* LBM 208. These strains were isolated from different tissue lesions of *Ilex paraguariensis* St. Hil (data not shown). Microorganisms were maintained at 4°C in Potato Dextrose Agar (PDA).

### Fungal inhibition assay

Dual culture assays were performed to evaluate the antagonism of *Trichoderma* strains against several phytopathogenic fungi following the methodology described by Desai et al. (2002), with modifications. Mycelial plugs of 5 mm diameter of *Trichoderma* and phytopathogen were placed on the same dish with PDA medium at 7 cm from each other. Paired cultures were incubated at 28 ± 1°C under constant light. Dishes inoculated only with test pathogens served as controls. Three replications of each plate were done. Radial pathogen growth was measured progressively. The inhibition of pathogenic fungal growth by the antagonist was evaluated quantitatively by means of the inhibition rate (IR). The IR was calculated after seven days of incubation using equation (1)

(1) $$IR\;=\;\frac{100\;\times \;(R_{2}-R_{1})}{R_{2}}$$

where IR is the percentage of reduction in mycelial growth of the phytopathogen, R1 is the average growth of pathogen in the treated plates, and R2 is the average growth of pathogen in the control plates. The *Trichoderma* strain that inhibited the pathogen growth 50% or more was considered an effective antagonist.

The antagonism index was calculated after 10 days of incubation using the Bell scale modified (Bell et al. 1982). A strain having an index of 3 or 4 is considered an effective antagonist. After 10 days of incubation, *Trichoderma* strains were tested for mycoparasitism and antibiosis against phytopathogens. For this purpose, the inhibition zone (confrontation zone) was cut using a sharp blade and transferred onto clean slides according to the methodology described by Kuzmanovska et al. (2018). Coverslips with a drop of lactophenol-cotton-blue (LCB) stain were mounted on the mycelia. Interactions such as circular winding (or “coiling”), side winding, and spores around the hyphae between the antagonist and the pathogen were observed under a light microscope.

### Diffusible and volatile metabolites

To verify diffusible metabolite production, we used the cellophane method described by Dennis and Webster (1971a), with modifications. Five-mm-diameter PDA plugs of *Trichoderma* were placed at the centre of Petri dishes containing cellophane sheets over PDA. After 5 days of incubation at 28 ± 1°C, the cellophane was removed, and a single 5-mm diameter mycelial plug of phytopathogen was placed at the centre of each dish. Each pathogen growing on PDA served as control. Plates were incubated at 28 ± 1°C for 5 days, and growth diameters were measured. Each condition was tested in triplicate; the results were expressed as percentage of growth inhibition calculated with Equation (1).

To evaluate the volatile metabolite production, quantitative and qualitative methods were used. The quantitative method was based on the methodology described by Dennis and Webster (1971b), with modifications. Plates with PDA were inoculated centrally with agar disks cut from stock cultures of *Trichoderma*. The lid of each dish was replaced by a bottom containing PDA inoculated with a phytopathogen strain. The two dishes were taped together and sealed with parafilm. The pathogen growth on PDA served as control. Plates were incubated at 28 ± 1°C under constant light for 7 days, and the radial pathogen growth was measured progressively. The percentage of growth inhibition was calculated with Equation (1). Test plates and control plates were set up in triplicate.

We followed the Strobel et al. (2001) methodology, with modifications, for qualitative determination. An agar strip of 2.5 cm wide was first removed from the centre of a Petri plate of PDA. A PDA plug with a 7-day-old mycelium of phytopathogenic fungi was placed on one side of the plate confronted with a plug of a 7-day-old mycelium of *Trichoderma* strain on the other side. Petri plates with phytopathogenic fungi growing alone were used as controls. Petri dishes were incubated at 28 ± 1°C under constant light for 7 days. At the end of the assay, the control plates were contrasted with the plates containing *Trichoderma* species and pathogenic strains.

### Qualitative determination of enzymatic activity, siderophore production, and phosphate solubilisation

Endoglucanase, lipase, protease, and chitinase activity determinations were carried out on agar plates containing minimum media with different carbon sources. A 5-day-old plug with mycelium of *Trichoderma* species was inoculated in different media. Determinations were replicated three times for each *Trichoderma* strain.

To establish the strains with the best characteristics, the strains showing the best qualitative features were used in dendrograms in the Numerical Taxonomy System (NTSys) program (Rohlf 2004) where 1 (one) was considered a positive result and 0 (zero) a negative result. Clustering was performed using the unweighted pair group method of arithmetical averages (UPGMA) algorithm based on Jaccard’s coefficient.

#### Endoglucanase activity

A medium with carboxymethylcellulose (CMC) as carbon source was used to verify endoglucanase activity. Petri dishes were incubated with plugs of different *Trichoderma* species for 5 days at 28 ± 1°C under constant light. After incubation, plates were revealed with 0.1% Congo Red dye for 5 min and washed with 5 M NaCl and 0.1% (v v^−1^) acetic acid. A clear halo around the colony represented a positive result that indicated endoglucanase production.

#### Lipolytic activity

To evaluate lipolytic activity, we used the methodology described by Howe and Ward (1976). The composition medium was 5 g NaCl, 0.1 g CaCl_2_, 10 g peptone, 20 g agar, and 10 g Tween 80 (polyoxyethylene sorbitan monooleate) per litre distilled water. Plates with *Trichoderma* strains were incubated for 5 days at the previously mentioned conditions. The colony capable to hydrolyse Tween 80 was considered positive due to the presence of opaque precipitate around the colony.

#### Proteolytic activity

*Trichoderma* strains were grown on Petri dishes containing 50 g skim milk and 10 g agar per litre distilled water (Dunne et al. 1997). Protease activity was manifested by the presence of a clear zone around the colony after 48 h of incubation at 28 ± 1°C with constant light.

#### Chitinase activity

Chitin from shrimp shells (Sigma C-7170) was used to determine chitinase activity. *Trichoderma* spp. were grown on a solid medium with colloidal chitin as carbon source, prepared according to Shimahara and Takiguchi (1988). The medium contained 1.5 g colloidal chitin, 2.7 g K_2_HPO_4_, 0.7 g MgSO_4_.7H_2_O, 0.5 g NaCl, 0.5 g KCl, 0.13 g yeast extract, and 15 g agar per litre distilled water at pH 5.5. *Trichoderma* strains were inoculated and incubated for 5 days at 28 ± 1°C with constant light. A positive result was observed as a transparent halo around the colony.

#### Siderophore production

Siderophore production was determined by using two different methods. In the first method, chrome-azurol S- agar (Louden et al. 2011) modified to pH 6.0 was inoculated with *Trichoderma* strains. After five days of incubation at 28 ± 1°C with constant light, strains exhibiting an orange halo were considered as siderophore producers. In the second method, 8-hydroxyquinoline (50 mg L^−1^) was added to malt extract agar (Hoyos-Carvajal et al. 2009). The strains capable to grow on this medium after 5 days of incubation at 28 ± 1°C were considered positive for siderophore production.

#### Phosphate solubilisation

Phosphate solubilization was determined using the National Botanical Research Institute’s phosphate medium (NBRIP) (Nautiyal 1999) containing 5 g Ca_3_(PO_4_)_2_, 5 g MgCl_2_.6H_2_O, 0.25 g MgSO_4_.7H_2_O, 0.2 g KCl, 0.1 (NH_4_)_2_SO_4_, 14 g agar, and 10 g glucose per litre distilled water. Agar plates were inoculated with a plug of *Trichoderma* and were incubated for five days at 28 ± 1°C with constant light. The presence of clear zones around the colonies indicated phosphate solubilisation ability.

### Bio-inoculation assay on yerba mate seedlings

Assays in organic nursery were carried out in order to evaluate the effect of *Trichoderma* spp. on some growth parameters of yerba mate seedlings after inoculation with this fungus. The assays were performed in an organic nursery of yerba mate in Santo Pipó, Misiones, Argentina, from May to September 2016. One-year-old yerba mate seedlings with the most homogenous phenotype were selected from the seedbed to be transplanted according to Bergottini et al. (2015). Yerba mate plants were inoculated with 5 ml per pot of a suspension of 10^7^ spores ml^−1^ of each *Trichoderma* sp. at different times (Yedidia et al. 1999). The inoculations were conducted at 7, 30, and 60 days after the first inoculation. Controls were irrigated with the same volume with distilled sterile water.

The length of the aerial part, the diameter, and the number of leaves were measured at the beginning and at the end of the assay. The dry weight of root and aerial parts was measured at the end of the assay.

### Statistical analysis

Results from antagonism, diffusible and volatile metabolites, and *in vivo* assay tests were statistically analysed using one-way ANOVA at 95% confidence limit and Fisher test with 5% confidence using the Statgraphics Centurion XV version 15.2.06 program.

## Results

### Fungal inhibition assay

All the evaluated *Trichoderma* strains were capable to inhibit the growth of all phytopathogens by 40% or more. There were no significant differences (p > 0.05) between the percentage of growth inhibition of all *Trichoderma* strains and the ones of phytopathogenic strains (data not shown).

**Figure 1.**
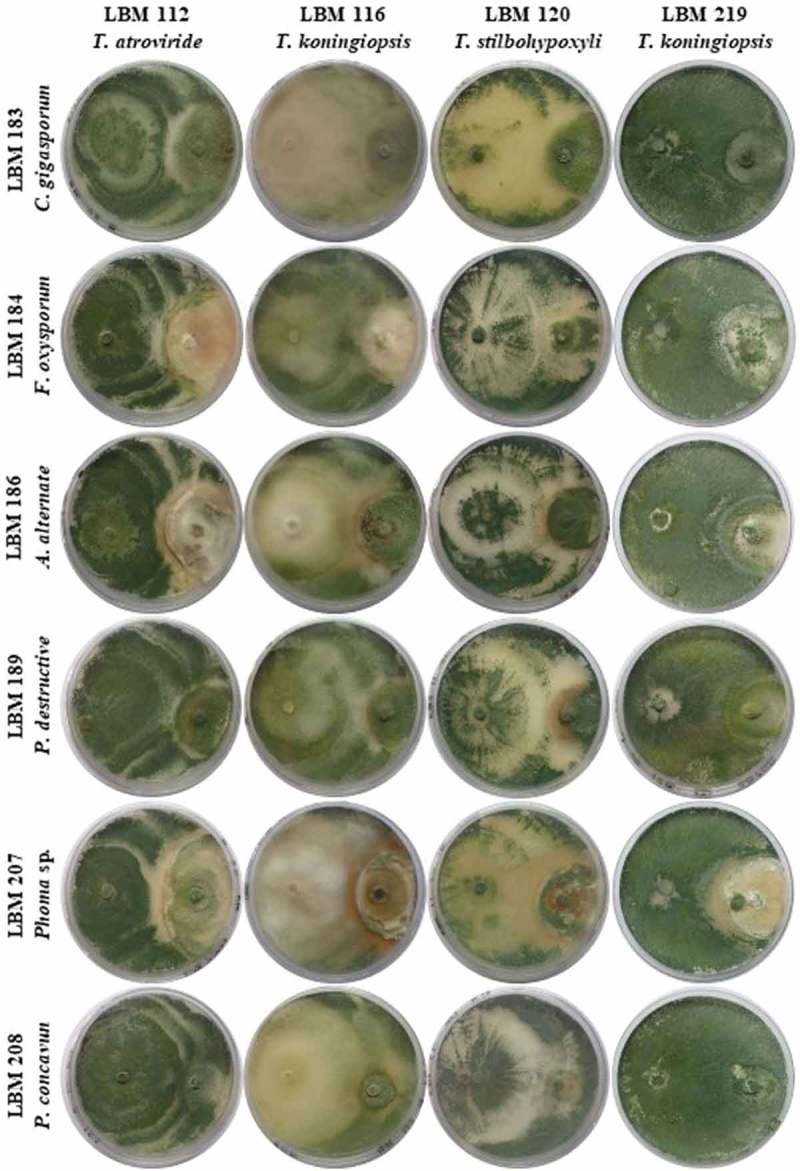
Photographs of PDA plates with *Trichoderma* and phytopathogenic strains after 10 days the assay started. *Trichoderma* strains were inoculated on the left side of the plate while phytopathogens were inoculated on the right side of the plate.

Figure 1 shows the plates with dual culture after 10 days of incubation; the Bell scale results are summarized in Table 1. Qualitative results after 10 days of incubation showed that *T. stilbohypoxyli* LBM 120 was capable to invade and reduce the growth of the six pathogens by more than 75%. *Phoma destructiva* LBM 189 and *P. concavum* LBM 208 were totally invaded by all the *Trichoderma s*trains used in the experiment with an antagonist index of 3 or 4 (Figure 1 and Table 1).

**Table 1.** Antagonist index after 10 days the assay started. Numbers correspond to the Bell scale modified.

Strain	Antagonist Index					
	LBM 183	LBM 184	LBM 186	LBM 189	LBM 207	LBM 208
LBM 112	I3	I1	I1	I4	I4	I4
LBM 116	I1	I1	I4	I4	I1	I4
LBM 120	I4	I4	I4	I3	I3	I3
*Trichoderma* sp.	I3	I3	I2	I4	I1	I4

**Figure 2.**
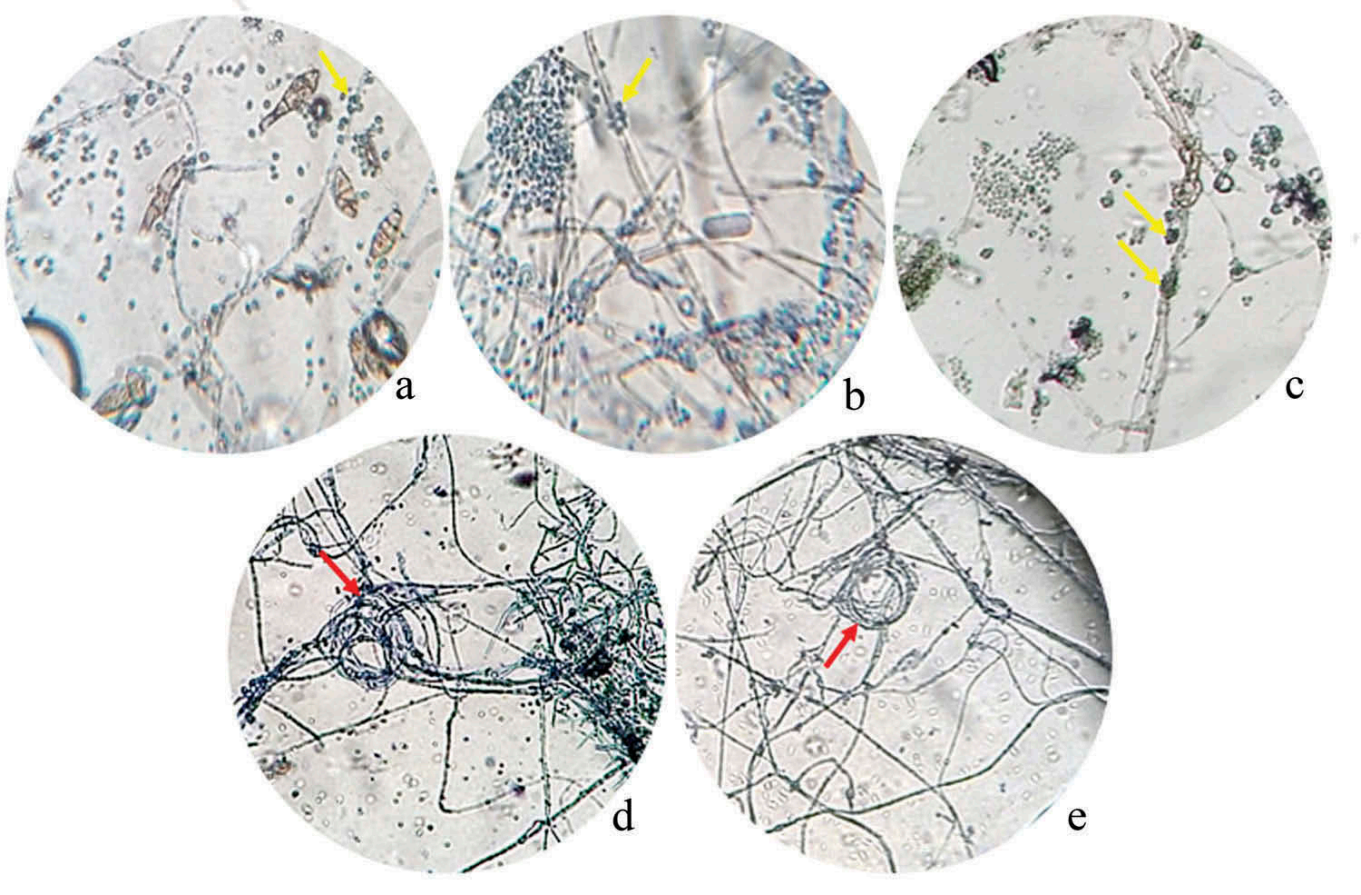
Photograph of interaction zones between *Trichoderma* and phytopathogenic strains after 10 days of incubation seen at a light microscope. A. *Trichoderma atroviride* LBM 112 interaction with *A. alternata* LBM 186 (40X); B. *Trichoderma koningiopsis* LBM 116 interaction with *C. gigasporum* LBM 184 (40X); C. *Trichoderma stilbohypoxyli* LBM 120 confronted with *Phoma* sp. LBM 207 (10X); D. *Trichoderma koningiopsis* LBM 219 confronted with *Phoma* sp. LBM 207 and E. *Trichoderma koningiopsis* LBM 219 confronted with *F. oxysporum* LBM 183 (10X). Yellow arrows show *Trichoderma* spores around pathogen hyphae and red arrows indicate the circular winding between *T. koningiopsis* LBM 219 and phytopathogens.

After 10 days post inoculation, the antagonism of *Trichoderma* spp. against phytopathogenic strains was observed as a dual culture with a light microscope (Figure 2). Light micrographs revealed two modes of antagonism by *Trichoderma* spp. against phytopathogenic strains. When *T. atroviride* LBM 112 was confronted with *A. alternata* LBM 186, *T. koningiopsis* LBM 116 was confronted with *C. gigasporum* LBM 183, and *T. stilbohypoxyli* LBM 120 was confronted with *Phoma* sp. LBM 207, we observed phytopathogenic hyphae surrounded by spore aggregate antagonists. We also detected that the hyphae of *Trichoderma* were intact (Figure 2(a–c), respectively). *Trichoderma koningiopsis* LBM 219 against both *Phoma* sp. LBM 207 and *F. oxysporum* LBM184 showed a circular winding pattern (Figure 2(d,e)) in the interaction zone.

### Diffusible and volatile metabolites

**Figure 3.**
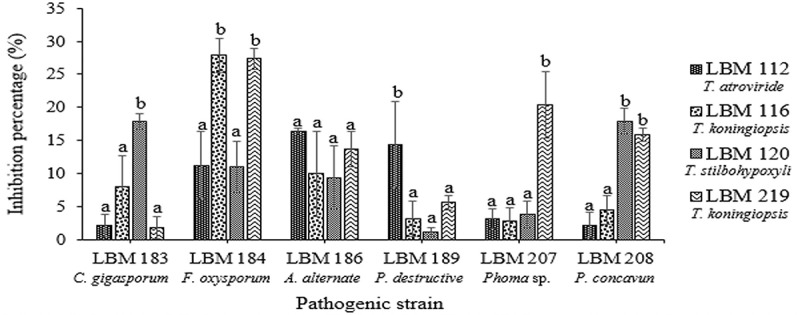
Percentage of phytopathogen growth inhibition by diffusible metabolites produced by *Trichoderma* strains. Standard error is represented with bars. The letter above the bars indicates homogenous group formation.

The results of the determination of diffusible metabolites showed that the growth of *C. gigasporum* LBM 183, *P. destructive* LBM 189, and *Phoma* sp. LBM 207 were inhibited by 15% or more by *T. stilbohypoxyli* LBM 120 (p = 0.0126), *T. atroviride* LBM 112 (p = 0.0052), and *T. koningiopsis* LBM 219 (p = 0.021), respectively. The growth of *F. oxysporum* LBM 184 was reduced in more than 25% by *T. koningiopsis* LBM 116 and LBM 219 (p = 0.0026). The growth of *A. alternate* LBM186 was inhibited by more than 15% by all *Trichoderma* strains without significant differences (p = 0.4087) whereas *T. stilbohypoxyli* LBM120 and *T. koningiopsis* LBM 219 were capable to inhibit the growth of *P. concavun* LBM 208 in more than 15% (p = 0.021). All pathogens were inhibited in more than 20% for at least one *Trichoderma* strain (Figure 3).

**Figure 4.**
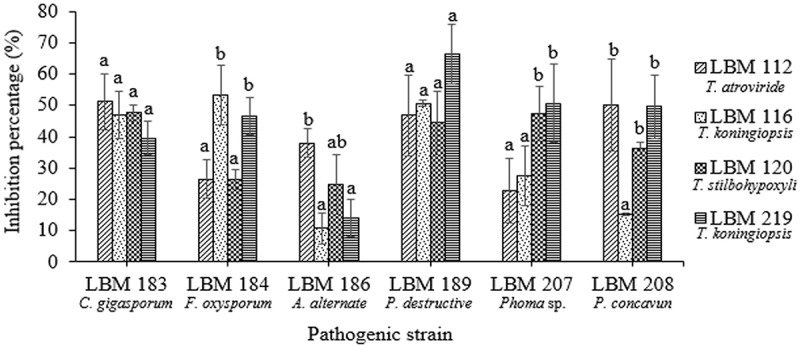
Percentage of phytopathogen growth inhibition by volatile metabolites produced by *Trichoderma* strains. Standard error is represented with bars. The letter above the bars indicates homogenous group formation.

**Figure 5.**
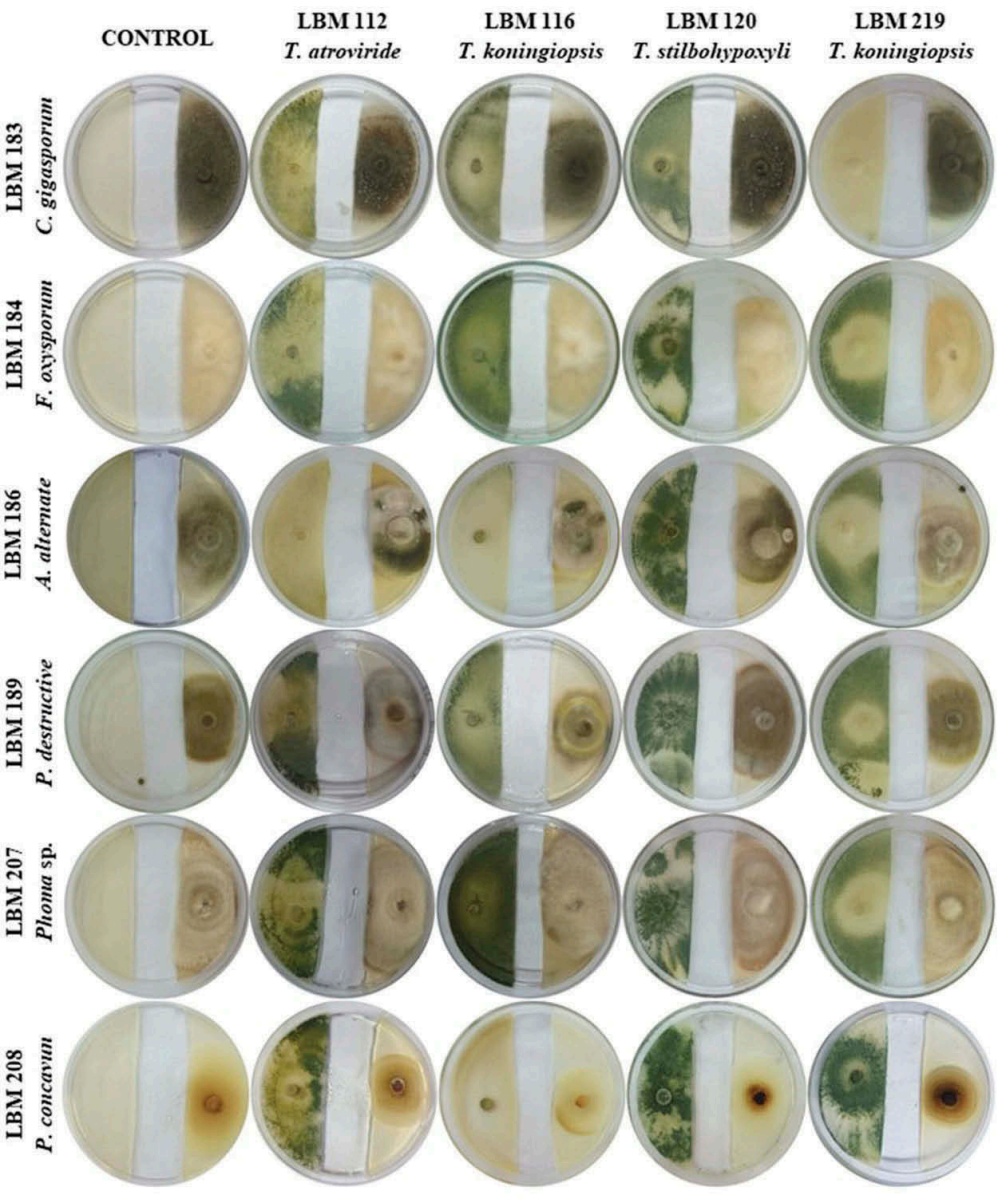
Photographs of the qualitative method of volatile metabolites production after seven days the assay started. The letter above the bars indicates homogenous group formation. *Trichoderma* strains were inoculated on the left side of the plate while phytopathogens were inoculated on the right side of the plate. Plate controls were only inoculated on the right side with pathogen strains.

The two methods used to determine volatile metabolites verified that all *Trichoderma* strains inhibited the growth of phytopathogenic fungi (Figures 4 and 5). *Fusarium oxysporum* LBM 184 growth was inhibited up to 46% by T. *koningiopsis* LBM 116 and *T. koningiopsis* LBM 219. These inhibition percentages differ significantly (p = 0.0027) with the results obtained for *T. atroviride* LBM 112 and *T. stilbohypoxyli* LBM 120. There were no significant differences between the inhibition percentages of all *Trichoderma* strains against *C. gigasporum* LBM 183 (p = 0.2544) and *P. destructiva* LBM 189 (p = 0.1441); both pathogens were inhibited up to 50% by volatile metabolites produced by all *Trichoderma* strains. The growth of *A. alternata* LBM 186 was reduced in more than 40% by *T. atroviride* LBM 112 (p = 0.0246). The growth of *Phoma* sp. LBM 207 was inhibited in more than 45% by *T. stilbohypoxyli* LBM 120 and *T. koningiopsis* LBM 219 with significant differences (p = 0.0247) from other *Trichoderma* spp. against the same phytopathogen. *Pilidium concavum* LBM 208 was inhibited in more than 36% by *T. atroviride* LBM 112, *T. stilbohypoxyli* LBM 120, and *T. koningiopsis* LBM 219 without significant differences (p = 0.0052), but *T. koningiopsis* LBM 116 only inhibited it in 15% (Figure 4).

### Qualitative determination of enzymatic activity, siderophore production, and phosphate solubilisation

**Figure 6.**
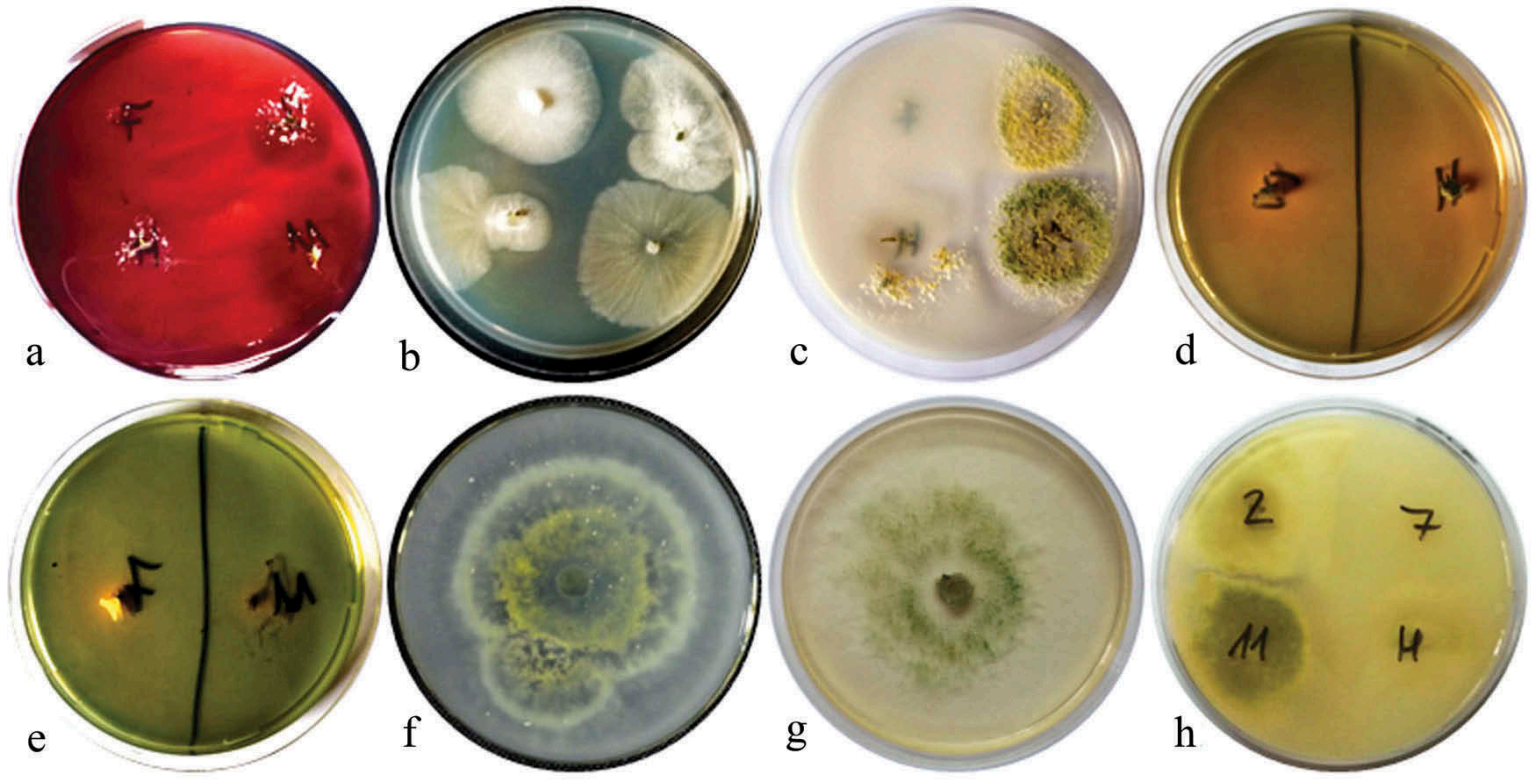
Plates show the results obtained from enzymatic determination, phosphate solubilisation and siderophore production. A. Endoglucanase activity*. B. Lipolytic activity*. C. Proteolytic activity*. D. Siderophore 1 production for *T. atroviride* LBM 112 and *T. koningiopsis* LBM 219. E. Siderophore 1 production for *T. koningiopsis* LBM 116 and *T. stilbohypoxyli* LBM 120. F. Positive results of chitinase activity. G. Siderophore 2 production as positive result. H. Phosphate solubilisation, 2: *Trichoderma atroviride* LBM 112, 7: *Trichoderma koningiopsis* LBM 116, 11: *Trichoderma stilbohypoxyli* LBM 120 and H: *Trichoderma koningiopsis* LBM 219. *The order of the strains on Petri plates is: *Trichoderma koningiopsis* LBM 116 at the top, on the left-hand side; *T. atroviride* LBM 112 at the top, on the right-hand side; *T. koningiopsis* at the bottom, on the left-hand side, *T.stilbohypoxyli* LBM 120 at the bottom, on the right-hand side.

**Figure 7.**
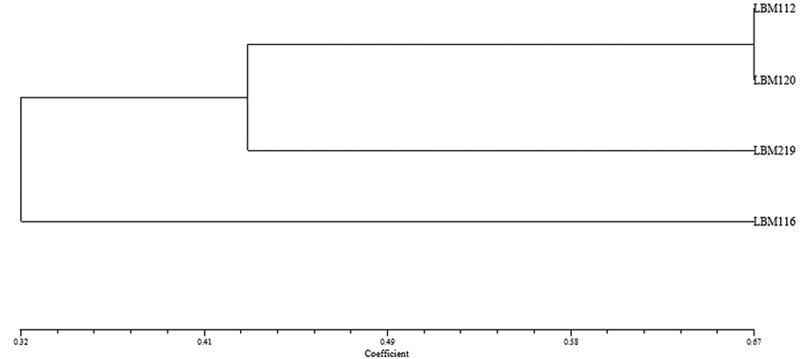
Dendrogram obtained with NTSys program using UPGMA and Jacard´s correlation.

*Trichoderma atroviride* LBM 112 and *T. stilbohypoxyli* LBM 120 showed positive results for endoglucanase, protease, chitinase, and phosphate solubilisation. *Trichoderma atroviride* LBM 112 was positive for siderophore production on CAS medium. However, *T. stilbohypoxyli* LBM 120 was positive for siderophore production using 8-hydroxyquinoline. *Trichoderma koningiopsis* LBM 116 was positive only for siderophore on CAS medium and chitinase activity whereas *T. koningiopsis* LBM 219 showed negative results in endoglucanase production and phosphate solubilisation (Figure 6). These results can be observed in the dendrogram shown in Figure 7, where *T. atroviride* LBM 112 and *T. stilbohypoxyli* LBM 120 were clustered in the same group, away from other *Trichoderma* strains.

### Bio-inoculation assay on yerba mate seedlings

**Figure 8.**
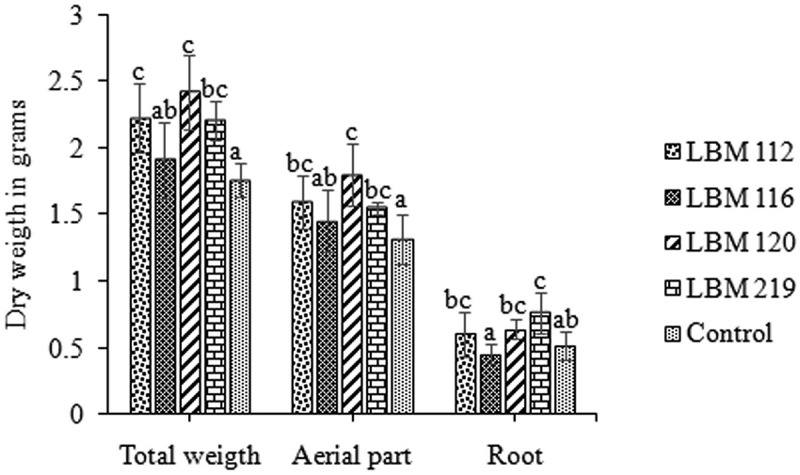
*In vivo* assay results. Representation of total, aerial part and root dry weight in grams. Standard error is represented with bars. The letter above the bars indicates homogenous group formation.

Native bio-inoculants had a highly significant positive effect on the growth of yerba mate seedlings in soil (Figure 8). *Trichoderma atroviride* LBM 112, *T. stilbohypoxyli* LBM 120, and *T. koningiopsis* LBM 219 produced a significant (p = 0.0016) increase on the dry weight of the total and aerial parts in contrast with uninoculated controls. The increases in aerial part mass were 28% for *T. atroviride* LBM 112, 49% for *T. stilbohypoxyli* LBM 120, and 24% for *T. koningiopsis* LBM 219; the total dry weight increases were 47%, 66%, and 46%, respectively. *Trichoderma koningiopsis* LBM 219 significantly (p = 0.0042) increased root dry weight by 25% in contrast with uninoculated controls. There were no significant differences in aerial part length, diameter, and leaf number among treatments at the beginning and at the end of the assay (data not shown).

## Discussion

*Trichoderma* presents different mechanisms to inhibit pathogen growth, which consist primarily of mycoparasitism, direct competition for space or nutrient, and production of metabolites or antibiotic (Howell 2003; Ezziyyani et al. 2004; Harman et al. 2004). Mycoparasitism is a complex process that involves the tropic growth of the biocontrol agent towards the target fungi, the lectin-mediated coiling of attachment of *Trichoderma* hyphae to the pathogen, and the attack and dissolution of the target fungus cell wall by the activity of enzymes, which may be associated with the physical penetration of the cell wall (Harman 2000).

According to our results, *Trichoderma* strains evaluated in this study displayed different interactions between the pathogen and the antagonists. *Trichoderma atroviride* LBM 112, *T. koningiopsis* LBM 116, and *T. stilbohypoxyli* LBM 120 showed high antagonism indexes, with hyphaes of phytopathogens surrounded by spore aggregate antagonists. These results can be correlated with the intense sporulation observed macroscopically and the detected production of enzymes. Amira et al. (2017) had observed this interaction among *Trichoderma harzianum* Ths97 and *Fusarium solani*. However, restricted by technical limits, no germination was clearly discernable after adhesion in their *in vitro* experiment.

On the other hand, we observed the development of helicoidal-shaped hyphae by *T. koningiopsis* LBM 219 around the pathogenic hyphae (or “coiling”). These coiled cell structures are commonly seen in various mycopathosystems (Moraga-Suazo et al. 2011; Amira et al. 2017).

As previously mentioned, *Trichoderma* spp. produce numerous biologically active compounds, including cell wall degrading enzymes (CWDEs) and secondary metabolites such as diffusible and volatile organic compounds (VOCs) (Vinale et al. 2008). *Trichoderma* VOCs would weaken the cell walls of phytopathogens, which could then be further affected by hydrolytic enzymes. (Moya et al. 2018). In our study, we observed that all phytopathogens were inhibited in more than 20% and more than 35% for at least one *Trichoderma* species on the determination of diffusible and volatile metabolites. The ability to produce volatile compounds was demonstrated in all the tested *Trichoderma* spp., suggesting the potential of these microorganisms as biocontrollers and biofertilizers. Our results suggest that the production of *Trichoderma* metabolites is related to their ability to invade and reduce the growth of pathogens and to the type of pathogens they are exposed to. It is known that the presence of other microorganisms enhances growth and induces the activation of genes related to parasitism and competition in several *Trichoderma* species (Atanasova et al. 2013). The control efficacy varies with the *Trichoderma* species and the target diseases (Harman et al. 2004).

In this context, the strain LBM 120 grew profusely on dual culture assays to inhibit growth of the six phytopathogens and produced hydrolytic enzymes, mainly chitinases, endoglucanases, and proteases, along with diffusible and volatile metabolites with the potential to destabilise the membrane systems. Similar results were demonstrated by Sharma et al. (2017) in antagonism assays between *T. velutinum* ACR-P1 and several phytopathogens.

Moreover, Kotasthane et al. (2015) suggested that several metabolic factors such as phosphate solubilisation and siderophore and auxin productions may be responsible for growth regulation in different agricultural and vegetable crops. Some *Trichoderma* spp. can penetrate and live endophytically within plant roots like mycorrhizal fungi (Kleifeld and Chet 1992); this lifestyle is common for Hypocreales (Zhang et al. 2018). In soil, various plant nutrients undergo complex transitions from soluble to insoluble forms that strongly influence their accessibility and absorption by roots (Harman et al. 2004). *Trichoderma asperellum* strain CHF 78 has several plant growth-promoting traits, such as the phosphate-solubilising ability and the production of siderophores, and could significantly increase plant dry weight (Li et al. 2018). Similarly, in our experiments, LBM 112 and LBM 120 showed positive results in at least one of the techniques tested for siderophore production and were able to solubilised phosphate; in greenhouse conditions, LBM 112 and LBM 120 had significant differences when contrasting the control with the aerial part and total dry weight of yerba mate plants.

On the other hand, we observed that LBM 219 was the only strain that showed positive siderophore production on both CAS and 8-hydroxyquinoline medium and showed significant differences in root dry weight contrasted with controls. These findings suggest that increased iron uptake by yerba mate plants promoted by the production of both siderophores LBM 219 was probably associated with enhanced dry root weight.

In previous studies, Bergottini et al. (2015) reported that three endophytic bacteria isolated from yerba mate root produced a highly significant increase in yerba mate biomass yield in soil in comparison to the non-native PGPR strain *A. brasilense* 245, which points out the importance of using native strains as effective bio-inoculants (Fages and Arsac 1991). Moreover, there are many studies on *Trichoderma* spp. showing their capacity to promote plant growth on cucumber, cacao, tomato, and other crops (Yedidia et al. 2001; Bae et al. 2009; Macías-Rodríguez et al. 2018) but, to our knowledge, the present report is the first one showing the native *Trichoderma* effect on yerba mate crops.

## Conclusion

Native *Trichoderma* strains from Misiones soil are promising fungi to improve the development of yerba mate crops. The strains tested in this work were able to inhibit pathogen growth using different mechanism involved in biological control and PGP properties such as the production of diffusible and volatile metabolites, the secretion of hydrolytic enzymes, the production of siderophores, and the solubilisation of phosphates.

The results from *in vitro* and *in vivo* assays allowed us to conclude that *T. stilbohypoxyli* LBM 120 and *T. koningiopsis* LBM 219 were the most promising microorganisms as alternatives for their application on yerba mate crops. We conclude that native *Trichoderma* strains isolated from Misiones soils could be a sustainable solution to improve yerba mate yield.

## References

[CIT0001] Amira MB, Lopez D, Mohamed AT, Khouaja A, Chaar H, Fumanal B, Gousset-Duponta A, Bonhommee L, Labela P, Goupila P, et al 2017 Beneficial effect of *Trichoderma harzianum* strain Ths97 in biocontrolling *Fusarium solani* causal agent of root rot disease in olive trees. Biol Control. 110:70–78.

[CIT0002] Atanasova L, Le Crom S, Gruber S, Coulpier F, Seidl-Seiboth V, Kubicek CP, Druzhinina IS. 2013 Comparative transcriptomics reveals different strategies of *Trichoderma* mycoparasitism. BMC Genomics. 14:121. 23432824 10.1186/1471-2164-14-121 PMC3599271

[CIT0003] Bae H, Sicher RC, Kim MS, Kim SH, Strem MD, Melnick RL, Bailey BA. 2009 The beneficial endophyte *Trichoderma hamatum* isolate DIS 219b promotes growth and delays the onset of the drought response in *Theobroma cacao*. J Exp Bot. 60(11):3279–3295. 19564160 10.1093/jxb/erp165 PMC2718224

[CIT0004] Bell DK, Wells HD, Markham CR 1982 *In vitro* antagonism of *Trichoderma* species against six fungal plant pathogens. Phytopathology. 72(4):379–382.

[CIT0005] Bergottini VM, Otegui MB, Sosa DA, Zapata PD, Mulot M, Rebord M, Zopfi J, Wiss F, Benrey B, Junier P 2015 Bio-inoculation of yerba mate seedlings (*Ilex paraguariensis* St. Hil.) with native plant growth-promoting rhizobacteria: a sustainable alternative to improve crop yield. Biol Fert Soils. 53(6):749–755.

[CIT0006] Dennis C, Webster J 1971a Antagonistic properties of species groups of *Trichoderma*: II. Production of non-volatile antibiotics. T Brit Mycol Soc. 57(1):25–39.

[CIT0007] Dennis C, Webster J 1971b Antagonistic properties of species groups of *Trichoderma*: II. Production of volatile antibiotics. T Brit Mycol Soc. 57(1):41–48.

[CIT0008] Desai S, Reddy MS, Kloepper JW 2002 Comprehensive testing of biocontrol agents In: Gnanamanickam SS, editor. Biological control of crop diseases. Boca Raton: CRC Press; p. 387–420.

[CIT0009] Dunne C, Crowley JJ, Moënne-Loccoz Y, Dowling DN, Bruijn S, O‘Gara F 1997 Biological control of *Pythium ultimum* by *Stenotrophomonas maltophilia* W81 is mediated by an extracellular proteolytic activity. Microbiology. 143:3921–3931. 10.1099/00221287-143-12-3921 33657716

[CIT0010] Eibl B, Fernández R, Kozarik JC, Lupi A, Montagnini F, Nozzi D 2000 Agroforestry systems with *Ilex paraguariensis* (American holly or yerba mate) and native timber trees on small farms in Misiones, Argentina. Agroforest Syst. 4:1–8.

[CIT0011] Ezziyyani M, Pérez C, Sid A, Requena ME, Candela ME 2004 *Trichoderma harzianum* como biofungicida para el biocontrol de *Phytophthora capsici* en plantas de pimiento (*Capsicum annuum* L.). An Biol. 26:35–45.

[CIT0012] Fages J, Arsac JF 1991 Sunflower inoculation with *Azospirillum* and other plant growth promoting rhizobacteria. Plant Soil. 137:87–90.

[CIT0013] Gravel V, Antoun H, Tweddell RJ 2007 Growth stimulation and fruit yield improvement of greenhouse tomato plants by inoculation with *Pseudomonas putida* or *Trichoderma atroviride*: possible role of indole acetic acid (IAA). Soil Biol Biochem. 3:1968–1977.

[CIT0014] Harman GE 2000 Myths and dogmas of biocontrol: changes in perceptions derived from research on *Trichoderma harzianum* T-22. Plant Dis. 84:377–393. 30841158 10.1094/PDIS.2000.84.4.377

[CIT0015] Harman GE, Howell CR, Viterbo A, Chet I, Lorito M 2004 *Trichoderma* species-opportunistic, avirulent plant symbionts. Nat Rev Microbiol. 2:43–56. 15035008 10.1038/nrmicro797

[CIT0016] Howe TGB, Ward JM 1976 The Utilization of Tween 80 as Carbon Source by *Pseudomonas B*. J G Microbiol. 92:234–235. 10.1099/00221287-92-1-234 812951

[CIT0017] Howell CR 2003 Mechanisms employed by *Trichoderma* species in the biological control of plant diseases: the history and evolution of current concepts. Plant Dis. 87:4–10. 30812698 10.1094/PDIS.2003.87.1.4

[CIT0018] Hoyos-Carvajal L, Orduz S, Bissett J 2009 Growth stimulation in bean (*Phaseolus vulgaris* L.) by *Trichoderma*. Biol Control. 51:409–416.

[CIT0019] Ilany T, Ashton M, Montagnini F, Martinez C 2010 Using agroforestry to improve soil fertility: effects of intercropping on *Ilex paraguariensis* (yerba mate) plantations with *Araucaria angustifolia*. Agrofor Syst. 80:399–409.

[CIT0020] Kleifeld O, Chet I 1992 *Trichoderma harzianum*—interaction with plants and effect on growth response. Plant Soil. 144:267.

[CIT0021] Kotasthane A, Agrawal T, Kushwah R 2015 In-vitro antagonism of *Trichoderma* spp. against *Sclerotium rolfsii* and *Rhizoctonia solani* and their response towards growth of cucumber, bottle gourd and bitter gourd. Eur J Plant Pathol. 141(3):523–543.

[CIT0022] Kuzmanovska B, Rusevski R, Jankulovska M, Oreshkovikj KB 2018 Antagonistic activity of *Trichoderma asperellum* and *Trichoderma harzianum* against genetically diverse *Botrytis cinerea* isolates. Chil J Agr Res. 78(3):391–399.

[CIT0023] Li YT, Hwang SG, Huang YM, Huang CH 2018 Effects of *Trichoderma asperellum* on nutrient uptake and *Fusarium* wilt of tomato. Crop Protect. 110:275–282.

[CIT0024] Louden BC, Harmann D, Lynne AM 2011 Tips and tools use of blue agar CAS assay for siderophore detection. J Microbiol Biol Educ. 51:51–53. 10.1128/jmbe.v12i1.249 PMC3577196 23653742

[CIT0025] Macías-Rodríguez L, Guzmán-Gómez A, García-Juárez P, Contreras-Cornejo HÁ 2018 *Trichoderma atroviride* promotes tomato development and alters the root exudation of carbohydrates, which stimulates fungal growth and the biocontrol of the phytopathogen *Phytophthora cinnamomi* in a tripartite interaction system. FEMS Microbiol Ecol. 94(9):Fiy137. 10.1093/femsec/fiy137 30010859

[CIT0026] Moraga-Suazo P, Opazo A, Zaldúa S, González G, Sanfuentes E 2011 Evaluation of *Trichoderma* spp. and *Clonostachys* spp. strains to control *Fusarium circinatum* in *Pinus radiata* Seedlings. Chilean J Agric Res. 71:412–417.

[CIT0027] Moya P, Girotti JR, Toledo A, Sisterna MN 2018 Antifungal activity of *Trichoderma* VOCs against *Pyrenophora teres*, the causal agent of barley net blotch. J Plant Protect Res. 58:45–53.

[CIT0028] Nautiyal CS 1999 An efficient microbiological growth medium for screening phosphate solubilizing microorganisms. FEMS Microbiol Lett. 170(1):265–270. 9919677 10.1111/j.1574-6968.1999.tb13383.x

[CIT0029] Poletto I, Brião MMF, Ceconi DE, Santin D, Deconto WMN, Blume E 2006 Zoning and identification of *Fusarium* spp. causing root rot in erva-mate plantings (*Ilex paraguariensis* A. St.-Hil.) in the Valley region of Taquari, RS. Ciênc Florest. 16:1–10.

[CIT0030] Prat Kricun SD, Belingheri LD 2003 Cosecha tradicional de la yerba mate [Traditional crop of yerba mate]. Cerro Azul: INTA, Estacion Experimental Agropecuaria; p. 12.

[CIT0031] Rohlf FJ 2004 NTSYS-pc numerical taxonomy and multivariate analysis system version 2.2. Exeter software Applied biostatics, New York.

[CIT0032] Sharma R, Magotra A, Manhasa RS, Chaubey A 2017 Antagonistic potential of a psychrotrophic fungus: *trichoderma velutinum* ACR-P1. Biol Control. 115:12–17.

[CIT0033] Shimahara K, Takiguchi Y 1988 Preparation of crustacean chitin. Method Enzymol. 161:417–423.

[CIT0034] Strobel A, Dirkse E, Sears J, Markworth C 2001 Volatile antimicrobials from *Muscodor albus*, a novel endophytic fungus. Microbiology. 147:2943–2950. 11700345 10.1099/00221287-147-11-2943

[CIT0035] Vinale F, Sivasithamparam K, Ghisalberti EL, Marra R, Woo SL, Lorito M 2008 *Trichoderma*–plant–pathogen interactions. Soil Biol Biochem. 40:1–10.

[CIT0036] Yedidia I, Benhamou N, Chet I 1999 Induction of defense responses in cucumber plants (*Cucumissativus* L.) by the biocontrol agent *Trichoderma harzianum*. Appl Environ Microb. 65:1061–1070. 10.1128/aem.65.3.1061-1070.1999 PMC91145 10049864

[CIT0037] Yedidia I, Srivastva AK, Kapulnik Y, Chet I 2001 Effect of *Trichoderma harzianum* on microelement concentrations and increased growth of cucumber plants. Plant Soil. 235(2):235–242.

[CIT0038] Zhang W, Zhang X, Li K, Wang C, Cai L, Zhuang W, Xiang M, Liu X 2018 Introgression and gene family contraction drive the evolution of lifestyle and host shifts of hypocrealean fungi. Mycol. 9:176–188. 10.1080/21501203.2018.1478333 PMC6115877 30181924

